# Cancer Risk and Eicosanoid Production: Interaction between the Protective Effect of Long Chain Omega-3 Polyunsaturated Fatty Acid Intake and Genotype

**DOI:** 10.3390/jcm5020025

**Published:** 2016-02-15

**Authors:** Georgia Lenihan-Geels, Karen S. Bishop, Lynnette R. Ferguson

**Affiliations:** 1 Wageningen University and Research Centre, 6708 PB Wageningen, the Netherlands; 2 Auckland Cancer Society Research Centre, University of Auckland; Private Bag 92019, Auckland 1142, New Zealand; k.bishop@auckland.ac.nz (K.S.B.); l.ferguson@auckland.ac.nz (L.R.F.)

**Keywords:** long chain omega-3 polyunsaturated fatty acid, cancer, single nucleotide polymorphism, eicosanoids, genotype

## Abstract

Dietary inclusion of fish and fish supplements as a means to improve cancer prognosis and prevent tumour growth is largely controversial. Long chain omega-3 polyunsaturated fatty acids (LC*n*-3 PUFA), eicosapentaenoic acid and docosahexaenoic acid, may modulate the production of inflammatory eicosanoids, thereby influencing local inflammatory status, which is important in cancer development. Although *in vitro* studies have demonstrated inhibition of tumour cell growth and proliferation by LC*n*-3 PUFA, results from human studies have been mainly inconsistent. Genes involved in the desaturation of fatty acids, as well as the genes encoding enzymes responsible for eicosanoid production, are known to be implicated in tumour development. This review discusses the current evidence for an interaction between genetic polymorphisms and dietary LC*n*-3 PUFA in the risk for breast, prostate and colorectal cancers, in regards to inflammation and eicosanoid synthesis.

## 1. Introduction

Cancer is a multifactorial, widely spread, variable and largely non-communicable disease, affecting populations in all parts of the world. Currently, some of the most significant cancers throughout the Western world include breast, colorectal and prostate cancers [1]. The role of nutrition in cancer risk and development is becoming increasingly recognised, particularly in regards to dietary intake of fresh fruit and vegetables, meat and meat products, and fish or fish oils, which may be related to their effects on inflammatory processes [2,3,4,5,6]. Intake of animal sources of fat, saturated and trans-unsaturated fatty acids are associated with all-cause mortality and death due to colorectal, breast and prostate cancers. On the other hand, plant based oils and fish oils are associated with a decrease in the risk and death due to the aforementioned cancers [7,8,9].

**Figure 1:**
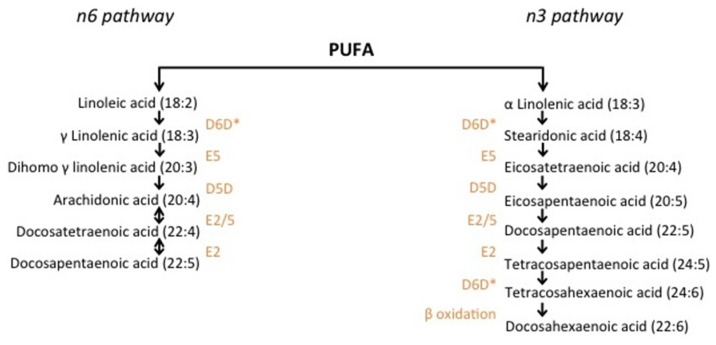
Synthesis of polyunsaturated fatty acids through the *n*-3 and *n*-6 pathways. Polyunsaturated fatty acid elongation from ALA and LA begins with desaturation by the D6D enzyme. Subsequent elongation and desaturation by the corresponding enzymes (orange) generates longer chain PUFA such as AA and EPA. *n*-3 and *n*-6 PUFA compete for the D6D, E5, D5D and E2 enzymes [11,12,13,14,15]. D5D: Delta 5 desaturase; D6D: Delta 6 desaturase; E: Elongase.*: Rate limiting step.

The predominant omega-6 (*n*-6) and omega-3 (*n*-3) fatty acids (FA) in the typical Western diet are linoleic acid (LA) (18:2*n*-6) and alpha-linolenic acid (ALA) (18:3*n*-3), respectively, known as the essential fatty acids. Through elongation and desaturation, these FA are converted to longer and more desaturated FAs via the *n*-6 and *n*-3 pathways (Figure 1). However, the conversion of LA and ALA to longer-chain FAs is limited by the enzymatic capacity of the desaturases, as well as dietary levels of LA and ALA, which compete for the same enzymes. For example, the conversion of ALA to eicosapentaenoic acid (EPA) (20:5*n*-3) ranges from between 0.2% and 21% [10].

**Figure 2:**
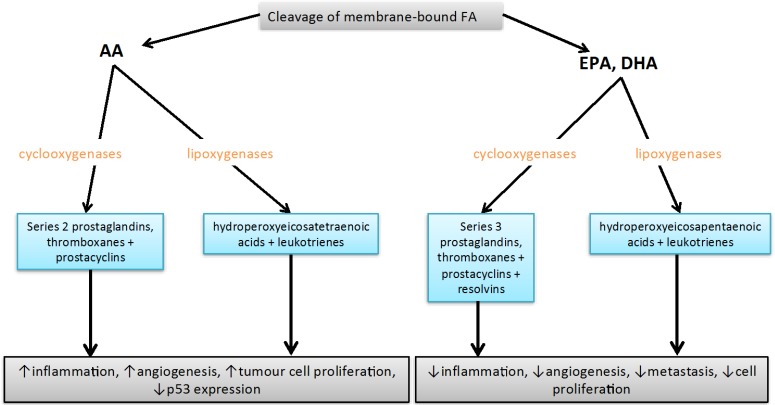
Effects of eicosanoids derived from AA and EPA/DHA. Cyclooxygenases and lipoxygenases act on AA, EPA and DHA to synthesise a range of different eicosanoids during an inflammatory response. AA-derived eicosanoids often generate pro-inflammatory compounds that enhance tumour growth, while EPA/DHA-derived eicosanoids often have anti-inflammatory properties and inhibit tumour growth [21,22]. FA: fatty acids; AA: arachidonic acid; EPA: eicosapentanoic acid; DHA: docosahexanoic acid.

Long chain (LC) polyunsaturated fatty acids (PUFA) play a significant role in inflammatory processes, as they act as precursors for inflammatory mediators called eicosanoids. Eicosanoids are potent signaling molecules synthesized during inflammation and include leukotrienes, thromboxanes and prostaglandins [16]. A diverse set of enzymes are responsible for the synthesis of eicosanoids from PUFA, some of which are outlined in Figure 2. Cyclooxygenases catalyse the formation of series 2 and series 3 prostaglandins and thromboxanes, while lipoxygenases synthesise lipoxins and leukotrienes, which are further metabolized by glutathione transferases [16]. Additionally, dietary EPA and docosahexaenoic acid (DHA) are precursors for mainly anti-inflammatory eicosanoids, while arachidonic acid (AA) (20:4*n*-6) is a precursor for mainly pro-inflammatory compounds and is in competition with EPA for eicosanoid production. Furthermore, the ratio of AA to EPA/DHA in cell membranes is thought to be informative in regards to inflammatory status. In fact, studies clearly show lower levels of circulating pro-inflammatory compounds such as cytokines and adhesion molecules with higher levels of membrane-bound and free EPA and DHA [17,18,19,20].

Inflammation is a key event in the development of tumours and is known to promote tumour growth, angiogenesis and metastasis [23]. For example, the metabolism of AA to pro-inflammatory eicosanoids is characteristic of some colorectal and breast cancer cells [24,25,26]. Therefore, dietary LC*n*-3 PUFA intake is of great interest in the prevention and treatment of these cancers and as agents in reducing inflammation, although the topic remains largely controversial [9,27,28,29]. Discrepancies in both observational and experimental data may arise from multiple sources including: heterogeneity of cancers; confounding in epidemiological data; environmental contaminants, particularly from LC*n*-3 PUFA-rich marine sources; accuracy of dietary intake data; bioavailability; and/or genetic variation [9,12,30,31,32,33]. One challenging aspect in cancer epidemiology is that any factor reducing cancer risk will usually promote life-expectancy, which in itself is a risk for cancer [34].

It has come to light that efficiency of conversion of LA and ALA to LC PUFA is partially determined by the genotype of the *fatty acid desaturase (FADS)* family of genes, which code for the delta-5 and delta-6 desaturases that catalyse the rate limiting steps of the *n*-3 and *n*-6 pathways, which may therefore impact downstream eicosanoid production [9,14,35,36,37,38]. Interestingly, single nucleotide polymorphisms (SNPs) found in other genes, for example *cytochrome c oxidase (COX)* and *arachidonate lipoxygenase (ALOX)*, may also influence levels of eicosanoids produced from EPA and AA [26,39]. *COX* and *ALOX* genes code for the cyclooxygenase and lipoxygenase enzymes, respectively, and are responsible for generating a range of eicosanoid mediators [16] (Figure 2). Consequently, both levels of dietary fatty acids and variation at the *FADS, COX* and *ALOX* loci may impact inflammatory processes and carcinogenesis.

There is increasing evidence to support the view that LC*n*-3 PUFA, specifically EPA and DHA, inhibit the growth of colorectal, breast and prostate cancer cell lines [27,40,41,42] and inhibit tumour growth in animal models [43,44,45]. Current evidence in humans is less clear and epidemiological data is largely inconsistent [9,27,28]. As inflammation is a predominant hallmark in many cancers, the relationship between inflammation and dietary LC*n*-3 PUFA is of high interest. Furthermore, the impact of genetic polymorphisms on the production of eicosanoids is important to consider. In this review, we discuss the interaction between LC*n*-3 PUFA and genotype, related to eicosanoid production, which may have an impact on the development and progression of cancers. The genes or family of genes under consideration include the *FADS* genes involved in the desaturation of LC*n*-3 PUFA [46], the *glutathione S-transferase (GST)* family of genes involved in oxidative stress and inflammation [47], and the *ALOX* and *COX* genes that generate pro- and anti-inflammatory mediators [48].

## 2. Methods

Articles utilised in this review were selected using the PubMed and Google Scholar databases. Key words used in the searches included: eicosanoid/s; polymorphism/s; cancer; dietary; polyunsaturated fatty acid; omega-3. One of these words must have also been present: prostate OR breast OR colorectal OR colon OR rectal AND FADS/fatty acid desaturase OR COX/cyclooxygenase OR ALOX/lipoxygenase OR GST/glutathione transferase. Included articles focused on human studies only. Exclusion criteria were: review articles; articles in any language other than English; articles older than 1990; articles focused on another disease other than breast, prostate or colorectal cancer; and articles lacking data on diet specific to PUFA or fish. Following searches of combinations of the above keywords, a total number of 417 articles were found. The titles of these 417 articles were read and after applying the exclusion criteria, 56 articles were selected for the next stage. Adjusting to the same exclusion criteria left a total number of 10 studies, which are summarised in Table 1.

**Table 1:** Polymorphisms associated with LC*n*-3 PUFA intake and cancer risk.

Reference	*n*	Subjects/methods	Exposure Measurement (PC; CC, CS)	Intervention (RCT)	Cancer type	Gene/s	Locus	Effect
*Al-Hilal et al.*, *2013* [14]	367	6-month RCT/M + F, 45–70 year		EPA + DHA; 0.45, 0.9 or 1.9 g/day		*FADS1 + FADS2*	rs174537	↓D5D activity associated with T variant allele; ↑D5D activity in TG, TT with increasing doses; no association for D6D
*Fradet et al.*, *2009* [12]	Control 478; case 466	CC/M, mean age 65 year	FFQ		Prostate	*COX2*	rs4648310	G allele correlated with aggressive PCa when *n*-3 FA intake was low, and low risk with high intake
*Gago-Dominguez et al.*, *2004* [49]	Control 670; case 258	CC/F, 45–74 year	SQ FFQ		Breast	*GSTM1, GSTT1, GSTP1*	multiple	Lower activity genotypes associated with higher BCa protection with ↑ intake of marine *n*-3 FA
*Habermann et al.*, *2013* [11]	Control 912; case 712	CC/M + F, 30–79 year	CARDIA questionnaire		Rectal	*ALOX12*	rs11571339	G allele associated with↓rectal cancer risk in those with low *n*-3 PUFA intake (NS)
*Habermann et al.*, *2013* [11]	Control 1900; case 1543	CC/M + F, 30–79 year	CARDIA questionnaire		Colon	*ALOX15*	rs11568131	AA genotype have ↓risk of colon cancer with↑ intakes of *n*-3 PUFA (NS)
*Habermann et al.*, *2013* [11]	Control 1900; case 1543	CC/M + F, 30–79 year	CARDIA questionnaire		Colon	*COX1*	rs10306110	Low EPA/DHA intake associated with higher colon cancer risk in variant allele carriers only
*Hedelin et al.*, *2006* [50]	Control 1130; case1499	CC/M, 35–79 year	FFQ		Prostate	*COX2*	rs5275	C allele at locus rs5275 correlated with ↓risk of PCa with high intake of fatty fish
*Hester et al.*, *2014* [13]	30	CS/Caucasian F, 21–65 year	Serum FA			*FADS1*	rs174537	T variant correlated with lower AA; GG genotype associated with ↑LTB_4_ + 5-HETE
*Hog et al.*, *2013* [37]	122	3 year PC/M, 35–59 year	Blood serum			*FADS1, FADS2 + FADS3*	rs174537 (FADS1); rs174575, rs2727270 (FADS2), rs1000778 (FADS3)	rs174537GG had ↑AA, AA/DGLA, DPA, LDL, oxLDL + ↓ETA. Rs17453 had↓AA, AA/DGLA, EPA, DPA, EPA/ALA + urinary PGF_2a_
*Poole et al.*, *2007* [51]	Control 626; case 716	CC/M + F, 30–74 year	FFQ		Colorectal	*COX1*	Phe17Leu	Modest↓risk of CRC for carriers of P17 with higher fish intake; L17 carriers have ↓risk of CRC with lower intake
*Poole et al., 2010* [52]	Control 582; case 483	CC/M + F, 30–74 year	FFQ		Colorectal	*PGES*	rs7873087	Carriers of T allele have ↓risk of CRC with ↑fish intake
*Poole et al.*, *2010* [52]	Control 582; case 483	CC/M + F, 30–74 year	FFQ		Colorectal	*EP4*	Val294Ile	Carriers of Ile variant showed correlation between ↑fish intake and ↑CRC risk
*Porenta et al.*, *2013* [53]	108	6-month RCT/CRC at risk M + F	2 day FR + 24 h recall	Healthy People 2010 diet or Mediterranean diet	Colon	*FADS* cluster	rs174556 and rs174561 in *FADS1*, rs383445 in *FADS2* and rs174537 of the *FADS1/2* intragenic region	Wild-type alleles associated with lower AA in colonic mucosa in persons on Mediterranean Diet

PC: Prospective cohort; CC: Case-control; CS: Cross-sectional; RCT: Randomized controlled trial; M: Males; F: Females; EPA: Eicosapentaenoic acid; DHA: Docosahexaenoic acid; D5D: Delta-5 desaturase; D6D: Delta-6 desaturase; FA: Fatty acid; LTB: Leukotriene B_4_; 5-HETE: 5-hydroxyeicosatetraenoic acid; CRC: Colorectal cancer; FR: Food recall; AA: Arachidonic acid; DGLA: Di-homo gamma linolenic acid; DPA: Docosapentaenoic acid; LDL: Low density lipoprotein; oxLDL: oxidized LDL; ETA: Eicosantetraenoic acid; ALA: Alpha-linolenic acid; PGF_2a_: Prostaglandin F_2a_; PUFA: Polyunsaturated fatty acid; NS: Non-significant; SQ: Semi-quantitative; FFQ: Food frequency questionnaire; BCa: Breast cancer; PCa: Prostate cancer.

## 3. The Role of Genetic Variation in Fatty Acid Desaturation

The *FADS* gene cluster is located on a highly polymorphic region of chromosome 11 and includes *FADS1* and *FADS2*, which encode delta 5 desaturase (D5D) and delta 6 desaturase (D6D), respectively [9]. These polymorphisms create a diverse set of haplotypes. The first demonstration of a relationship between *FADS* genotype and membrane-bound FAs was shown by Schaefer *et al.* (2006) [54] in serum phospholipids. Further evidence came from a study on infants, in which Danish infants carrying the *FADS* minor allele for locus rs1535 had a higher DHA level than those with the wild-type allele [55]. In contrast, those carrying the minor alleles of rs174448 (C) and rs174575 (G) had decreased DHA levels relative to wild-type [55]. Similarly, carriers of the T allele at rs174537 (in strong linkage disequilibrium with rs174546 and rs3834458) had lower levels of AA than the carriers of the G allele [37]. Additional examples have been presented by Al-Hilal *et al.* (2013) in which the minor allele of SNPs rs174537, rs174561 and rs3834458 correlate with higher amounts of ALA and lower levels of EPA, docosapentaenoic acid (DPA) and DHA, as well as lower activity of both D5D and D6D [14].

Of particular interest to the interplay with dietary LC*n*-3 PUFA, a 6-month intervention of an EPA/DHA supplement in individuals carrying a T allele at locus rs174537 showed rising activity of D5D with an increasing supplement dose [14]. Additionally, polymorphisms at *FADS* locus rs174546 correlated with serum triacylglycerides at baseline and 6 weeks following EPA/DHA supplementation [56]. At locus rs174537, the presence of a T-allele correlated with lower levels of AA, consistent with a similar study [37], and those carrying the GG genotype had higher levels of eicosanoids leukotriene B_4_ (LTB_4_) and 5-hydroxyeicosatetraenoic acid (5-HETE) [13]. LTB_4_ and 5-HETE are pro-inflammatory compounds synthesized from AA by the leukotriene synthase and 5-LOX enzymes, respectively [39]. Therefore, it is possible that levels of circulating eicosanoids may be modulated by the interplay of diet and genotype. If these individuals are at particular risk for cancer, it would be advisable to increase the intake of LC*n*-3 PUFA.

Studies on colonic mucosal fatty acid compositions have revealed a diet-genotype effect. Lower concentrations of AA were observed in subjects carrying major alleles within the *FADS* gene cluster (rs174556 and rs174561 in *FADS1*, rs383445 in *FADS2* and rs174537 of the *FADS1/2* intragenic region) when consuming a Mediterranean diet compared to a Healthy Eating diet, due to increases in AA levels within the Healthy Eating group [53]. The Mediterranean diet has been extensively studied with regards to its effect on cancers. This diet is traditionally high in fat, but low in LC*n*-6 PUFA and *trans* fatty acids, and is typically high in olive oil, fresh fruit and vegetables [57,58]. The Mediterranean diet used in an intervention by Porenta *et al.* (2013) [53] was also high in fish and flaxseed. Additional studies are required to confirm these results, as diets were not strictly controlled and sample size was relatively small. A summary of the interaction between LC*n*-3 PUFA on prostate, breast and colorectal cancers as modified by *FADS* genotype, is provided in Table 1, alongside additionally discussed genotypes.

## 4. Genetic Polymorphisms Modulate Leukotriene Synthesis in Cancer

### 4.1 Lipoxygenases

Leukotrienes are eicosanoid inflammatory mediators produced by the oxidation of AA, and are implicated in inflammation and cancer [59]. Leukotriene synthesis begins with the formation of hydroperoxyeicosatetranoic acid (5-HPETE) and hydroperoxyeicosapentaenoic acid (5-HPEPE) from AA and EPA, respectively, by the lipoxygenases [16]. Although not statistically significant, a lower risk for colon cancer was demonstrated in wild-type homozygous individuals at locus rs11568131 of *ALOX15* when consuming high amounts of fish, an association that was absent in those carrying the variant allele [11]. Carriers of a G minor allele at locus rs11571339 of the *ALOX12* gene showed a lower risk for rectal cancer in those with low *n*-3 PUFA intake compared to higher intakes [11]. However, G allele carriers with high intakes showed no increased risk compared to the homozygous major allele reference group. Despite also demonstrating no statistical significance, this finding is particularly interesting, as a lower LC*n*-3 PUFA intake would generate fewer anti-inflammatory compounds than a higher intake. Furthermore, G allele carriers with lower intakes of LA and total PUFA showed a similar pattern. Studies investigating the differences in activity of 12-lipoxygenase due to this polymorphism could help to explain these findings.

A recent meta-analysis found that polymorphisms in the *ALOX12* gene at the Gln261Arg locus may influence cancer risk in Asian populations but not in Caucasians [60]. Furthermore, carriers of the variant in homozygous or heterozygous form had an increased risk for breast cancer, also demonstrating differences across ethnic populations [61]. The same polymorphism also showed an association with risk of colorectal adenomas [62]. To our knowledge, the interplay of this polymorphism with EPA/DHA intake has not been previously explored and is worthy of further investigation.

### 4.2 Glutathione S-Transferases

The glutathione S-transferase (GST) enzymes implicated in various types of cancer are important for the detoxification of environmental pollutants and chemical carcinogens, and modulate signaling of pathways associated with cell proliferation, cell differentiation and apoptosis [47]. In addition, GSTs are involved in the synthesis of leukotrienes from 5-HPETE. Finally, GSTs are also important for the detoxification of reactive oxygen species [63] and may help protect against DNA damage [64].

Raised levels of anti-oxidants can help activate *GST* genes and this in turn may help to reduce the increased levels of DNA damage that are associated with prostate cancer [36,64]. *GST* phenotype (e.g., *GSTT1* null genotype) is associated with risk of prostate cancer in Caucasians but this does not hold true for other races [47]. Unfortunately, no evidence appears to be available with respect to the modification of this effect by fatty acids in prostate cancer. However, van Hemelrijck *et al.* (2012) [65] identified an association between prostate cancer and the intake of heterocyclic aromatic amines (HCAs) that was modified by the genotype of HCA-metabolizing enzymes (e.g., *MnSOD* rs4880 and *GPX4* rs713041). HCAs are mutagenic and are generated by cooking meat at high temperatures [66]. Meat is a common source of animal fat and the effect of some monounsaturated fatty acids (e.g., palmitic and stearic acids) as well as *n*-6 PUFA (e.g., AA) on prostate cancer may be confounded by the presence of HCAs. For this reason, we propose that while the genotype of HCA-metabolizing enzymes may appear to interact with type of fatty acid intake and prostate cancer risk, in fact it is the presence of HCAs that is interacting with genotype to influence disease risk.

In contrast, a clear association has been shown between the polymorphic *GST* genes, breast cancer and marine FA intake [49]. Women carrying variants resulting in higher activity of the GST enzymes show a correlation with marine *n*-3 PUFA intake and risk of breast cancer, in which lower intake demonstrates a higher risk compared to those with higher intakes of the same genotypes. These associations were found in Chinese and Singaporean women [49].

## 5. Prostaglandin Synthesis

Cyclooxygenase enzymes, also known as prostaglandin endoperoxide synthases, catalyse the rate-limited formation of inflammatory prostaglandins. Two isozymes (*COX1* and *COX2*) exist, both of which are associated with injury and inflammation and demonstrate different tissue expression patterns [12]. Increased expression of *COX2* leads to hyperproliferation of colon epithelial cells, a process which was decreased following the presence of EPA [67]. Furthermore, the inhibitory effects of non-steroidal anti-inflammatory drugs (NSAIDs) associated with colorectal cancer are thought to relate to their inhibitory activity at both *COX1* and *COX2* [68].

*In vitro* studies have shown inhibitory actions of LC*n*-3 PUFA on prostate cancer cell growth. In different prostate cancer cell models, namely LNCaP and PacMetUT1, DHA appeared to sensitise the cells by attenuating the NF-κB survival pathway that promotes cancer cell survival, resulting in decreased cancer cell survival [69]. On the other hand, NF-κB does not appear to be involved in the induction of *COX2* expression in the prostate cancer cells, PC3, treated with DHA and EPA [70]. In regards to human studies, five of sixteen SNPs found within the *COX2* region were tested in a Swedish population with and without prostate cancer, identifying a relationship between two SNPs and the presence of prostate cancer [50,71]. Subsequently, the same authors demonstrated that the presence of a C allele at locus rs5275 was significantly associated with a decreased risk of prostate cancer in men with a high intake of fatty fish [50]. Similarly, Fradet *et al.* (2009) [12] assessed diet alongside nine *COX2* SNPs in men diagnosed with aggressive prostate cancer and found that LC*n*-3 PUFA intake was strongly associated with a decreased risk of aggressive prostate cancer. This effect was modified by the rs4648310 SNP, such that the increased risk of aggressive prostate cancer associated with a low intake of *n*-3 PUFA in those with the G allele (odds ratio = 5.49) could be reversed by increasing *n*-3 PUFA intake [12]. Therefore, it is reasonable to say that carriers of a G allele at the rs4648310 locus could benefit from increasing LC*n*-3 PUFA intake.

*COX1* SNPs at the rs10306110 locus may modulate colon cancer risk. Habermann *et al.* (2013) [11] demonstrated an association between low LC*n*-3 PUFA intake and the variant allele, with an odds ratio of 1.56 and 1.62 for EPA and DHA, respectively. Total and monounsaturated fatty acid intake was associated with the variant allele at rs10306122 of *PTGS1*, the gene encoding *COX1*, and increased rectal cancer risk, although marine LC PUFA showed no effect [11].

The P17L polymorphism, leading to sequence changes within the signal peptide of *COX1*, was associated with risk of colorectal adenomas, in which higher fish intake in those homozygous for phenylalanine at position 17 had a modestly lower risk of adenomas with increasing fish intake [51]. Interestingly, those carrying at least one leucine at position 17 had a decreased risk of adenomas when consuming less fish per week [51]. Importantly, these individuals demonstrated a higher risk for colorectal cancer with increasing fish intake. This is a highly interesting finding which highlights the occurrence of inconsistencies in studies of cancer and LC*n*-3 PUFA and the importance of designing and performing studies that will provide clarity in this regard.

The same authors [51] then analysed the risk of colorectal adenomas between those in an assumed low risk group (high fish intake + NSAID use) and an assumed high risk group (low fish intake + no NSAID use) and variable intermediate groups, to assess the dual implications of both NSAID use and fish intake in the relationship between P17L polymorphisms and adenoma risk. PP homozygotes benefited from including more than 2 servings of fish per week as well as regular use of NSAIDs. However, those with PL and LL genotypes showed no statistically significant associations [51]. These findings are unexpected and it is necessary to replicate these investigations in larger studies with more detail on type of fish in the diet, as well as other dietary information.

Polymorphisms within the gene for *prostaglandin E_2_ synthase-1 (PGES)* also correlate with colorectal adenoma risk. *PGES* catalyses the formation of PGE_2_, a pro-inflammatory prostaglandin associated with increased cell proliferation [72,73]. Individuals carrying a T allele at rs7873087 had a lower risk for colorectal adenomas with increasing fish intake, whereas those homozygous for the A allele showed no significant association with fish intake [52]. Additional relationships were observed for polymorphisms within the *15-hydroxyprostaglandin dehydrogenase* gene and the *EP4 receptor* gene, which code for proteins responsible for the breakdown of PGE_2_ and the corresponding PGE_2_ receptor, respectively. These studies highlight the importance of inter individual differences in genes involved in the prostaglandin synthesis pathways from AA and EPA, and their complex association with colorectal cancer and fish intake. This relationship warrants further investigation.

Limitations in these studies include recall bias in the FFQ and diet diaries, which are commonly used in large studies such as cohorts or case-control designs due to lack of affordable and better alternatives. Furthermore, ethnicity must be adjusted for in studies, as ethnicity may influence the relationship between dietary *n*-3 PUFA, cancer risk and genotype, as highlighted earlier [60,61]. Additional factors potentially influencing the outcome of the studies reviewed herein, include the stage of the disease, as LC*n*-3 PUFA may interact differently with genotypes as the physiology of the tumour changes, and fish contaminants. Dioxins may increase cancer risk, which could generate substantial confounding [33]. In addition, it is important to note that this review highlights the current knowledge of the interplay between genes involved in eicosanoid synthesis only, and that there are a range of other genes that are likely to contribute to the relationship between cancer risk and LC*n*-3 PUFA intake, such as polymorphisms in DNA repair- and apoptosis-related genes [74].

## 6. Conclusions

The effects of LC*n*-3 PUFA on prostate, breast and colorectal cancer modified by genotype are presented in Table 1. It is clear that both dietary intake and polymorphisms of the *FADS* genes contribute to the concentrations of membrane-bound fatty acids such as EPA, DHA and AA. Although genetic variation within the *FADS* genes have not been directly associated with cancer, the effects on desaturase activity may influence the production of eicosanoids further downstream. Dietary LC*n*-3 PUFA (EPA, DPA and DHA) are inversely associated with aggressive prostate cancer [12] and prostate cancer risk. This protective effect can be modified by genotype including rs5275 [50] and rs4648310 [12] in *COX2*. On the other hand, the loss of expression of *FADS2*, in response to a mutation in *FAD2*, is associated with a more aggressive breast cancer tumour and reduced survival [9,75]. Breast cancer risk may also be modulated by dietary LC*n*-3 PUFA and activity of the GST enzymes [49]. Interestingly, an association between *ALOX12* polymorphisms and breast cancer, which was modified by ethnicity [60,61] could be further explored in regards to the relationship with LC*n*-3 PUFA intake. In regards to colon and rectal cancers, certain individuals may benefit largely from including LC*n*-3 PUFA in their diets while others do not, as demonstrated by polymorphisms in *ALOX12*, *ALOX15* and *PGES* genes [11,52]. Furthermore, there exists a positive association between increased risk of colorectal cancers and increased fish intake in some genotypes of the *COX1* gene, a relationship worthy of further investigation.

Compelling evidence from *in vivo* and *in vitro* studies has been presented on the inhibition of cancer progression. Here, evidence has been presented on the genotypic modification of response to LC*n*-3 PUFA and it is clear that we are on the brink of offering personalised nutritional advice with respect to these FAs. Such advice would ensure that people are correctly informed with respect to the types and amounts of LC*n*-3 PUFA they should consume in order to meet their specific requirements. In addition, further study could decipher the significance of the role of *n*-3 PUFA in cancer and inflammation, for example whether altered PUFA metabolism is a driver or a passenger in cancer [76].
